# The Reactome Pathway Knowledgebase

**DOI:** 10.1093/nar/gkx1132

**Published:** 2017-11-14

**Authors:** Antonio Fabregat, Steven Jupe, Lisa Matthews, Konstantinos Sidiropoulos, Marc Gillespie, Phani Garapati, Robin Haw, Bijay Jassal, Florian Korninger, Bruce May, Marija Milacic, Corina Duenas Roca, Karen Rothfels, Cristoffer Sevilla, Veronica Shamovsky, Solomon Shorser, Thawfeek Varusai, Guilherme Viteri, Joel Weiser, Guanming Wu, Lincoln Stein, Henning Hermjakob, Peter D’Eustachio

**Affiliations:** European Molecular Biology Laboratory, European Bioinformatics Institute (EMBL-EBI), Wellcome Genome Campus, Hinxton, Cambridgeshire CB10 1SD, UK; Open Targets, Wellcome Genome Campus, Hinxton, Cambridgeshire CB10 1SD, UK; NYU School of Medicine, New York, NY 10016, USA; Ontario Institute for Cancer Research, Toronto, ON, M5G 0A3, Canada; College of Pharmacy and Health Sciences, St. John's University, Queens, NY 11439, USA; Oregon Health Sciences University, Portland, OR 97239, USA; Department of Molecular Genetics, University of Toronto, Toronto, ON M5S 1A1, Canada; National Center for Protein Sciences, Beijing, China

## Abstract

The Reactome Knowledgebase (https://reactome.org) provides molecular details of signal transduction, transport, DNA replication, metabolism, and other cellular processes as an ordered network of molecular transformations—an extended version of a classic metabolic map, in a single consistent data model. Reactome functions both as an archive of biological processes and as a tool for discovering unexpected functional relationships in data such as gene expression profiles or somatic mutation catalogues from tumor cells. To support the continued brisk growth in the size and complexity of Reactome, we have implemented a graph database, improved performance of data analysis tools, and designed new data structures and strategies to boost diagram viewer performance. To make our website more accessible to human users, we have improved pathway display and navigation by implementing interactive Enhanced High Level Diagrams (EHLDs) with an associated icon library, and subpathway highlighting and zooming, in a simplified and reorganized web site with adaptive design. To encourage re-use of our content, we have enabled export of pathway diagrams as ‘PowerPoint’ files.

## INTRODUCTION

At the cellular level, life is a network of molecular reactions that include signal transduction, transport, DNA replication, protein synthesis, and intermediary metabolism. A variety of online resources capture aspects of this information at the level of individual reactions such as Rhea (1) or at the level of reaction sequences spanning various domains of biology such as KEGG (2), MetaCyc (3) or PANTHER (4). The Reactome Knowledgebase is distinctive in focusing its manual annotation effort on a single species, *Homo sapiens*, and applying a single consistent data model across all of these domains of biology. Processes are systematically described in molecular detail to generate an ordered network of molecular transformations, resulting in an extended version of a classic metabolic map (5). The Reactome Knowledgebase systematically links human proteins to their molecular functions, providing a resource that functions both as an archive of biological processes and as a tool for discovering unexpected functional relationships in data such as gene expression surveys or catalogs of somatic mutations in tumor cells.

Reactome (version 62—September 2017) has entries for 10 719 human genes, 53% of the 20 338 predicted human protein-coding genes (http://www.ensembl.org/Homo_sapiens/Info/Annotation), supporting the annotation of 24 704 specific forms of proteins distinguished by co- and post-translational modifications and subcellular localizations. These function with 1768 small molecules as substrates, catalysts, and regulators in 11 302 reactions annotated on the basis of data from 27 526 literature references. These tallies include 1334 mutant variants and their post-translationally modified forms derived from 285 gene products, used to annotate 906 disease-specific reactions, tagged with 294 Disease Ontology terms (6). These reactions form 2102 pathways (e.g. Interleukin-15 signaling; phosphatidylinositol phosphate metabolism; receptor-mediated mitophagy) grouped into 26 superpathways that correspond to domains of biology such as metabolism and signal transduction. Reactome's dataset continues to grow briskly, with 74 new human pathways added in the first three quarters of 2017.

Notable additions include extensive new annotations of cytokine signaling, including a comprehensive catalog of known interleukin signaling pathways. We have also revised and supplemented existing pathways, continuing to build our catalogs of signaling processes mediated by G protein-coupled receptors, of transport processes, and of metabolism. Notably, where our initial annotations in these domains centered on the most extensively studied, ‘textbook’ versions of pathways and molecules, we are now systematically adding proteins whose properties indicate closely-related biological roles, to increase the density and connectivity of the reaction network in Reactome that is available for visualization and computational analysis.

This growth has come at a cost. Our SBGN-based (7) scheme for representing pathways, implemented eight years ago, yields pathway diagrams that become cluttered and difficult for biologist users to navigate as we achieve more nearly complete annotations of the participants in a process and their functions, and our approximately 120 diagrams of superpathways are essentially lists of the names of component pathways, functional but uninformative and unappealing to biologist users. Meanwhile, this growth in the number and complexity of our annotations has made our relational data structure slow and unwieldy for handling complex queries and large scale data analyses.

We have addressed the computational aspects of these challenges by implementing a Neo4j graph database structure for our production web site, developing a new high-performance in-memory implementation of our core overrepresentation data analysis tool, and implementing a new Pathway Diagram Viewer to support faster data loading, diagram rendering and element seeking. To improve usability we have developed Enhanced High Level Diagrams (EHLDs) that combine an iconography familiar from textbooks and review articles with web functionality, to represent superpathways, and have added features to our pathway diagrams to improve their legibility. A redesigned web site with adaptive technology is accessible from tablets and mobile devices, and supports more intuitive navigation.

Here, we provide brief descriptions of these changes and their contributions to the usability of the Reactome data resources.

## IMPROVED PERFORMANCE AND SCALABILITY

### Implementation of a graph database

Relational databases work well to model and store complex pathway information and their engines have been optimized to provide efficient execution of global SQL queries that aggregate large amounts of data without requiring traversal operations. Reactome data, however, contain many relationships and thus many join tables, so queries generally require traversal operations resulting in degraded performance and long response times.

To preserve our well-established and well-tested tools for data annotation and internal storage while improving performance of our public resource, we continue to maintain the relational Reactome database but have developed a graph database batch importer to migrate content to a Neo4j graph database during each quarterly release. Third parties can directly use Cypher, a declarative, pattern matching query language specifically designed to retrieve data from graph data structures. A fully documented REST based web content service has been built on top of the graph-core to provide third party developers with programmatic access to the graph database. Stress-testing the graph and relational implementations of Reactome indicates approximately a 30-fold improvement in throughput for queries submitted to the graph implementation. Users can download the Reactome graph database and access the data through Cypher queries directly on their computers. In the first year that Reactome provided the graph database, there were 2385 downloads by 912 unique users, 118 of whom downloaded the graph database after each data release.

The Reactome graph database is freely available at: https://reactome.org/dev/graph-database. The API for the ContentService is available at https://reactome.org/ContentService with documentation and tutorials available at: https://reactome.org/dev/content-service. The Java source code is freely available at: https://github.com/reactome in the graph-core, graph-importer and content-service repositories. (See Table 1 for a summary of on-line resources discussed in this paper.)

**Table 1.** Reactome online resources

Home page	https://reactome.org
an introductory video for users	https://youtu.be/-skixrvI4nU
The Reactome graph database	https://reactome.org/dev/graph-database
API for the ContentService	https://reactome.org/ContentService
documentation and tutorials	https://reactome.org/dev/content-service
Java source code	https://github.com/reactome
includes graph-core, graph-importer, content-service and analysis-tools repositories	
EHLD source code	https://github.com/reactome-pwp/diagram
reusable stand-alone JavaScript EHLD viewer	https://reactome.org/dev/diagram/js
EHLD icon library	https://reactome.org/icon-lib
community contributions to the library	https://reactome.org/icon-info

### Improved performance of data analysis tools

Reactome provides an over-representation analysis tool (8) to support interpretation of expression data sets. The tool calculates whether a user-generated list of proteins, for example, ones whose expression is changed in response to a stress, contains more annotated to each Reactome pathway than would be expected by chance given the number of proteins in the set, the number annotated to the pathway, and the number annotated in all of Reactome. The Reactome implementation uses a hypergeometric distribution test to generate a probability score, which is corrected for false discovery rate using the Benjamani-Hochberg method (9,10). We have now developed a high-performance in-memory implementation that divides the method into four steps, each with a specific data structure to improve performance and minimize the memory footprint. First, each identifier in the user's sample is matched to an entity in Reactome using a radix tree as a lookup table. Second, a graph is used to model proteins, chemicals, their orthologs in other species and their composition in complexes and sets. The third and fourth steps aggregate the results and calculate the statistics, employing a double-linked tree. This implementation provides a stable, high performance pathway analysis service, enabling the analysis of genome-wide datasets within seconds, allowing interactive exploration and analysis of high throughput data. It is accessible on our web site both via the AnalysisService for programmatic access and a user submission interface integrated into the PathwayBrowser. All of its source code is freely available in the AnalysisTools repository in the Reactome GitHub (https://github.com/reactome/) (10).

### Data structures and strategies to boost diagram viewer performance

A new version of the Pathway Diagram Viewer (version 3) was implemented to provide faster data loading, diagram rendering and element seeking, with the goal of completing most user interactions in less than a second. Improvements include: (i) restructuring of the data format used to send the data from the server to the client from XML to JSON, (ii) using a graph data structure to store the pathway content on the client side, (iii) boosting the client content load strategy, (iv) implementing a multi-layer canvas approach and (v) utilizing a space partitioning data structure to store the elements to be rendered. Conversion from XML to JSON reduced diagram sizes only by about 20% but reduced processing and loading times by at least 65% over the full range of sizes.

## IMPROVED PATHWAY DISPLAY AND NAVIGATION

### Implementation of interactive Enhanced High Level Diagrams (EHLDs)

To improve the quality of the graphics used to represent pathways and to make pathway navigation easier, we have integrated three new features into the Reactome pathway browser: textbook-style EHLDs to represent superpathways such as signaling and immune function, a mechanism to highlight different subpathways with colored overlays in zoomed-out views of detailed pathway diagrams coupled to changes in amount of detail displayed as users zoom into and out of diagrams, and an option to export EHLDs, individual EHLD icons and pathway diagrams in editable, reusable forms (11).

**Figure 1.**
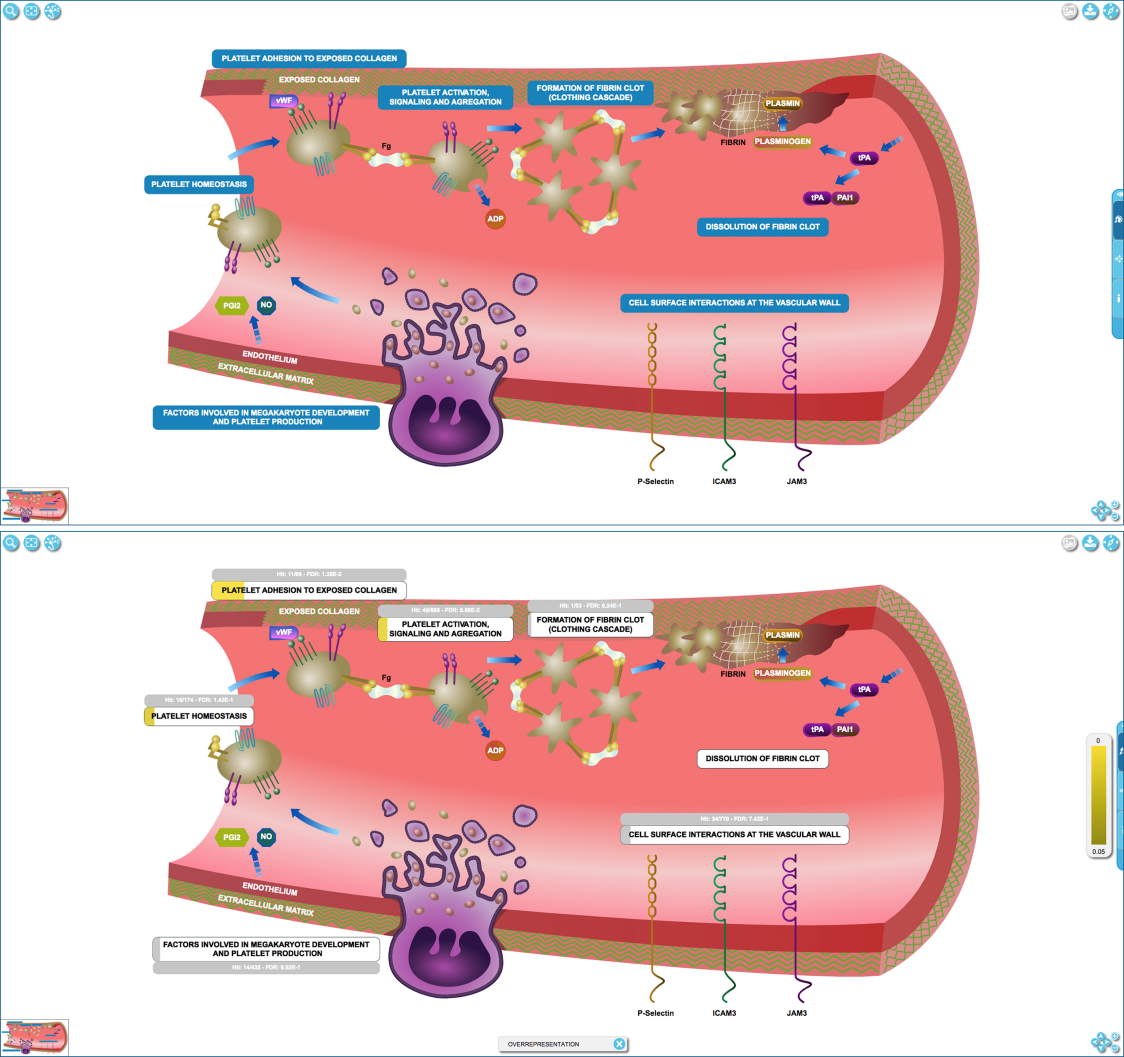
‘Hemostasis’ top-level pathway represented as an Entity High Level Diagram (EHLD) above, and with expression data analysis results overlaid, below, showing relative overexpression of gene products involved in platelet adhesion to exposed collagen.

EHLDs (Figure 1) resemble overviews of biological processes shown in textbooks or review articles, with a consistent iconography based on widely used simple diagrams of molecules, cellular structures, cells, and tissues, to make navigation intuitive and familiar. This design helps the user to recognize the process represented in an EHLD and select the appropriate region of the EHLD to navigate to the next more detailed level of the Reactome hierarchy, ultimately to arrive at a detailed pathway diagram that shows individual physical entities participating in individual reactions.

EHLDs were produced by a team of curators and illustrators who revised the Reactome event hierarchy as necessary and developed overview diagrams for the higher levels of the hierarchy. They developed a common style and iconography to enhance the ‘recognition effect’ and facilitate efficient navigation. Of the 120 high-level pathways represented as lists of subpathway names, 48 have been converted to EHLDs; conversion of the remainder should be complete by late in 2018.

The SVG format was selected for EHLDs because it allows object-based vector representation for easy editing, resolution-independent zooming, interaction features for richer interfaces, use of data in regular text format as a subset of an eXtensible Markup Language (XML) that can be easily handled by computer programs or text editors, and support by most popular web browsers and compatibility with most popular graphic software packages.

EHLDs have a limited number of entities, so the multilayered HTML5 Canvas approach implemented for detailed pathway diagrams (above) was not needed. Instead, for EHLDs, their simple overlay features and a rendering strategy where the flow of information does not depend on the level of zoom, allowed the adoption of an SVG renderer. This allowed developers to implement features such as overlays, zoom and translation by applying a series of filters and transformations.

The Reactome web interface now has an SVG rendering component that, beyond standard zooming and panning, allows specific regions of an EHLD to be highlighted, recognizes mouse click events on specific regions to allow navigation to subpathways and allows analysis results to be overlaid on it. All of the required technical annotations were included in the SVG files by using the database identifier attribute of each relevant diagram element. Any element with no technical annotation was considered a decoration, so EHLDs can accommodate placeholder icons for subpathways that are still in development. The code is part of the EHLD package available on the Reactome public GitHub repository (https://github.com/reactome-pwp/diagram). EHLDs are also available in the reusable stand-alone JavaScript diagram viewer (https://reactome.org/dev/diagram/js).

Pathway analysis results (1) can be overlaid on EHLDs. The results are displayed in the label of each subpathway, which is overlaid by a colored rectangular shape whose width and color represent the percentage of hit entities and the *P*-value, respectively (Figure 1).

EHLDs, including any analysis result overlay, can be easily downloaded and saved in SVG format. Users can edit the content of the exported files with commercial or open-source graphics applications. EHLDs use a consistent iconography that reuses glyphs when the same entity plays a role in more than one biological process. We have made a library of these graphical elements to provide them in SVG, PNG and EMF formats. The icon library is available at https://reactome.org/icon-lib and is distributed under the terms of the CC-BY license (https://creativecommons.org/licenses/by/4.0/) to facilitate the creation of uniform diagrams through the use of pre-existing glyphs and to offer these components to the community for reuse.

Researchers can use these icons to create their own illustrations to convey their findings and ideas, whether in a paper or a grant application. Additionally, we encourage the community to contribute new icons to extend this library (https://reactome.org/icon-info).

### Subpathway highlighting and zooming

**Figure 2.**
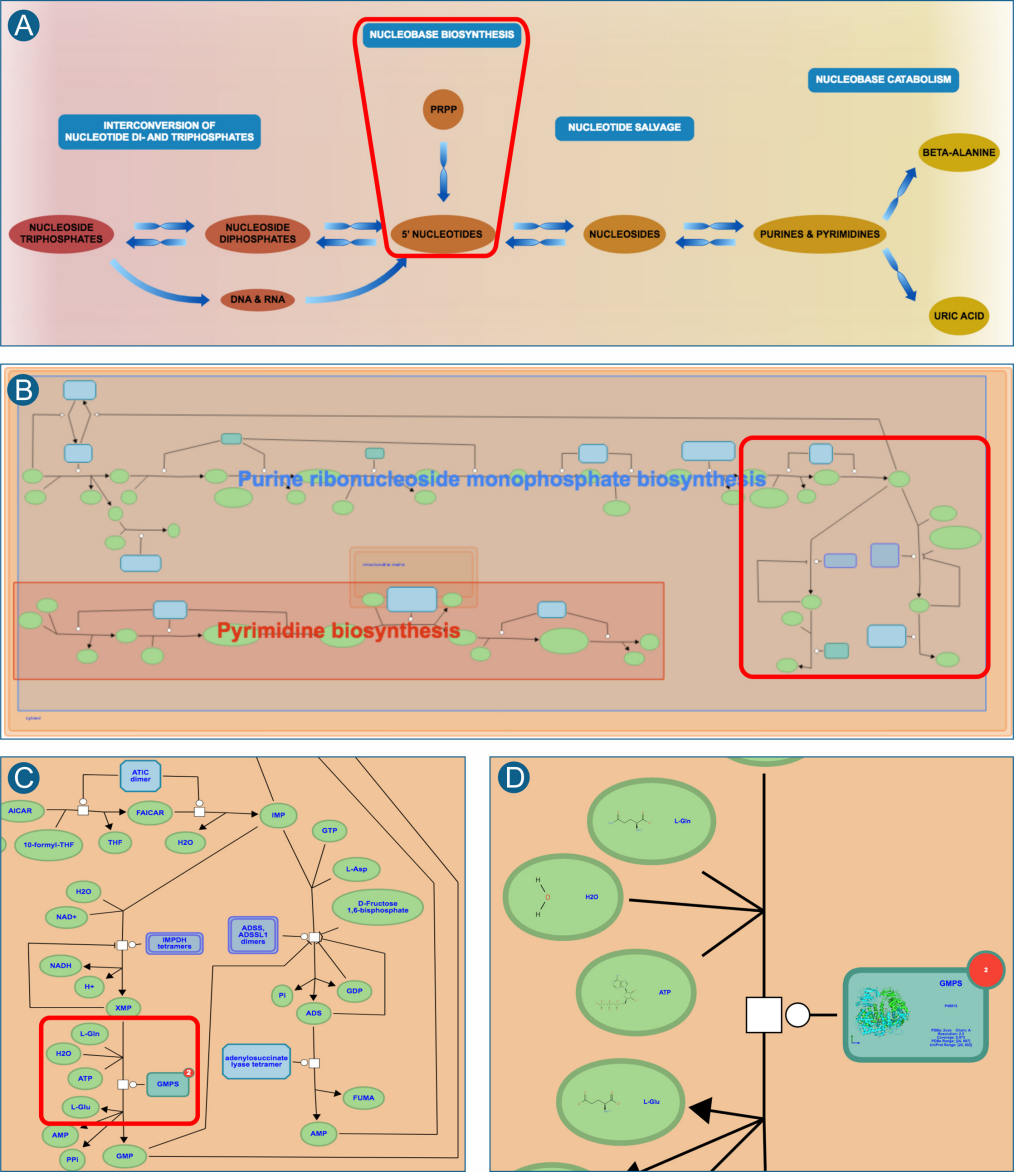
Pathway navigation with detail matched to zoom level. A user who selects the pathway ‘nucleotide metabolism’ is presented with an EHLD that shows the entire process with its major subpathways labeled (**A**). Double-clicking anywhere in the region of nucleobase biosynthesis (red box in A) yields a pathway diagram for that process, with its subpathways labeled and only its major components shown (**B**). As the user zooms in to view the last steps of purine biosynthesis (red box in B), housekeeping entities are revealed and names of all entities are displayed (**C**). Finally, as the user zooms in to a specific region of the pathway (red box in C), structures of small molecules and proteins are shown (**D**).

To make the navigation of complex SBGN-like pathway diagrams easier, the web display has been modified to make the amount and kind of information displayed dependent on zoom level, as shown in Figure 2. When a user enters a pathway diagram from the event hierarchy or a superpathway EHLD, only key components are shown, unlabeled, but with boxes superimposed to highlight pathway boundaries and show pathway names. As a user zooms further into the pathway more detail is shown until individual entities are shown as molecular structures. For fast loading and a consistent look and feel, the position and color of pathway boxes is calculated on the server side at database release time.

### Export of pathway diagrams as ‘PowerPoint’ files

Pathway diagrams have previously been available as static PNG images. To enable users to modify diagrams for their own use, we have developed a strategy to export a pathway diagrams from the Pathway Browser as an interconnected set of objects that adapt to layout changes so that when a glyph is moved in an exported diagram all connected objects follow. Our implementation (11) generates PPTX files on the server side, using the color profile selected by the user on the client side and uses Aspose.Slides (https://www.aspose.com/products/slides/java), a commercial JAVA API for reading, writing and manipulating PowerPoint documents, to store the content in PPTX format.

### A simplified and reorganized web site with adaptive design

**Figure 3.**
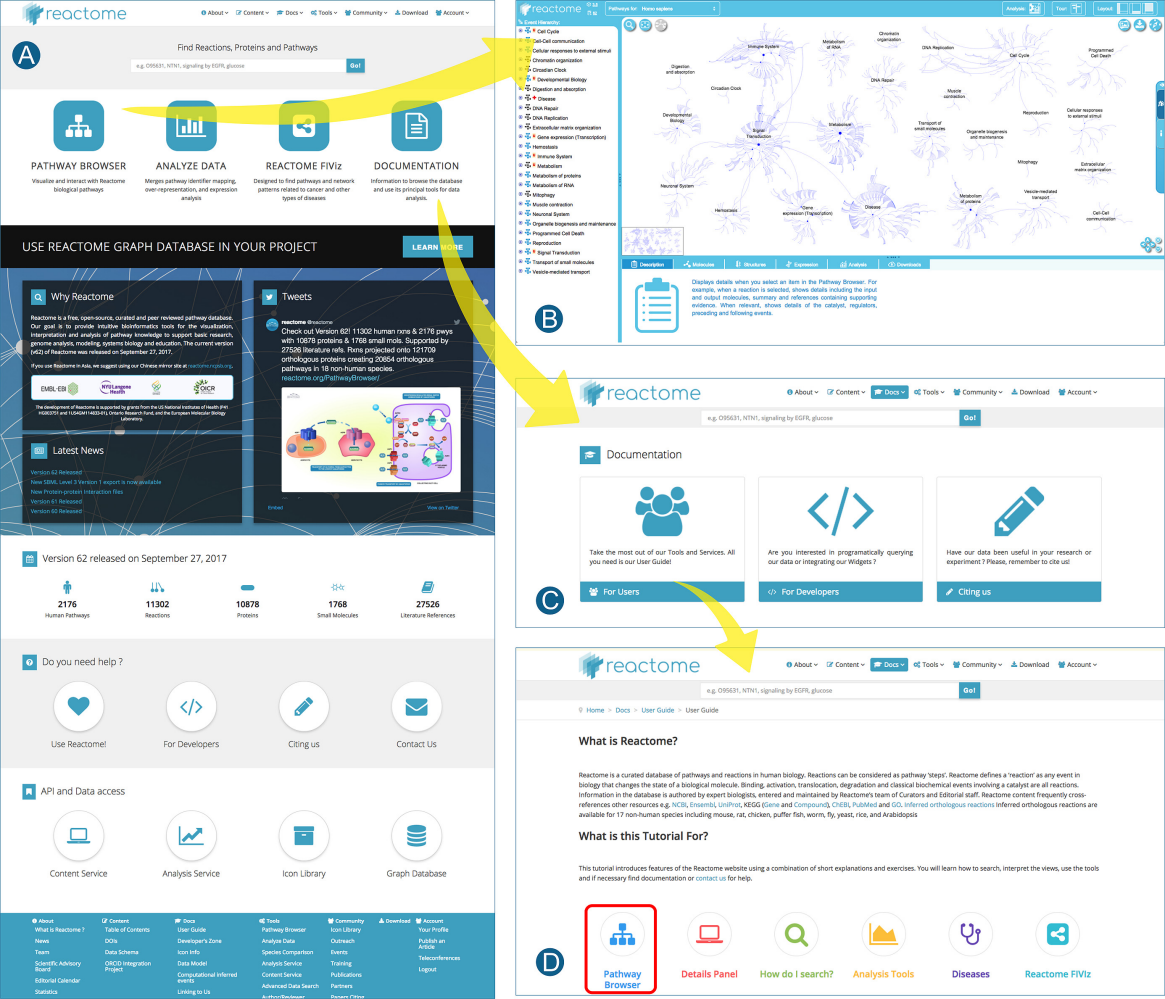
Reorganized Reactome website. From the simplified home page (**A**), a single step leads to the pathway browser (**B**), our main tool for data visualization and analysis for human users. Users needing help in navigating and interpreting the site can navigate in a single step to on-line documentation, organized by topic and including solved examples (**C**). The same documentation button also leads to pages for developers who want access to our analysis service, content service, graph database and widget to include Reactome pathway displays in their web applications (**D**).

We have redesigned our landing page and several key pages linked to it to simplify them and make the navigation to desired tools and documentation more straightforward (Figure 3). The new design includes adaptive features to make our content accessible from tablets and mobile devices. Documentation for biologist users and developers has been revised to bring it up to date and reorganized to make it more easily navigated.

A video that introduces the pathway browser and data analysis tools is available at https://youtu.be/-skixrvI4nU.

## CONCLUSIONS

Both the Reactome Knowledgebase and user needs for data visualization and analysis have grown in size and complexity, and will continue to grow briskly. We have addressed the computational aspects of this growth and change by implementing a Neo4j graph database structure for our production web site, developing a new high-performance in-memory implementation of our core overrepresentation data analysis tool, and implementing a new Pathway Diagram Viewer to support faster data loading, diagram rendering and element seeking. To improve usability we have developed Enhanced High Level Diagrams (EHLDs) that combine an iconography familiar from textbooks and review articles with web functionality, to represent superpathways, and have added features to our pathway diagrams to improve their legibility. A redesigned web site with adaptive technology is accessible from tablets and mobile devices, and supports more intuitive navigation.
